# Herring gulls respond to human gaze direction

**DOI:** 10.1098/rsbl.2019.0405

**Published:** 2019-08-07

**Authors:** Madeleine Goumas, Isabella Burns, Laura A. Kelley, Neeltje J. Boogert

**Affiliations:** Centre for Ecology and Conservation, University of Exeter, Penryn Campus, Treliever Road, Penryn TR10 9FE, UK

**Keywords:** gaze sensitivity, gaze aversion, herring gulls, human–wildlife conflict, kleptoparasitism

## Abstract

Human–wildlife conflict is one of the greatest threats to species populations worldwide. One species facing national declines in the UK is the herring gull (*Larus argentatus*), despite an increase in numbers in urban areas. Gulls in urban areas are often considered a nuisance owing to behaviours such as food-snatching. Whether urban gull feeding behaviour is influenced by human behavioural cues, such as gaze direction, remains unknown. We therefore measured the approach times of herring gulls to a food source placed in close proximity to an experimenter who either looked directly at the gull or looked away. We found that only 26% of targeted gulls would touch the food, suggesting that food-snatching is likely to be conducted by a minority of individuals. When gulls did touch the food, they took significantly longer to approach when the experimenter's gaze was directed towards them compared with directed away. However, inter-individual behaviour varied greatly, with some gulls approaching similarly quickly in both treatments, while others approached much more slowly when the experimenter was looking at them. These results indicate that reducing human–herring gull conflict may be possible through small changes in human behaviour, but will require consideration of behavioural differences between individual gulls.

## 1. Introduction

Interactions between humans and wildlife often have detrimental impacts on a wide variety of taxa, and human–wildlife conflict is a major cause of species declines and limited success of conservation efforts [1]. Intervention tends to focus on reducing negative effects on humans through managing wildlife populations. However, wildlife management is often ineffective [2], targets non-problem individuals or jeopardizes the conservation status of the targeted species [1]. It is increasingly being recognized that, rather than solely imposing controls on wildlife, changes in human behaviour could alleviate these conflicts while also benefiting conservation efforts [3].

Conflict between herring gulls (*Larus argentatus*) and humans is an ongoing source of debate and control measures. This species is on the UK's Red list of Birds of Conservation Concern as the British population decreased by 60% between 1969 and 2015 [4] owing to rapid anthropogenic change [5]. Despite their decreasing overall population size, increasing numbers of herring gulls are breeding in urban areas [5]; the traditional nesting sites of cliffs and islets have been exchanged for roofs, and marine prey is sometimes largely substituted with anthropogenic food [5].

As well as being scavengers and predators, herring gulls are kleptoparasites [6] and take food from both conspecifics and heterospecifics. Herring gulls in urban environments appear to have generalized their kleptoparasitic activities to target humans, resulting in numerous complaints to local authorities and increasing human–herring gull conflict [5]. Attempts to decrease this conflict have focused largely on lethal control or deterrents (e.g. birds of prey), which often prove costly and ineffective and ignore species- and individual-specific behaviour [5]. Understanding the behaviour of wildlife at both the population and individual level is important in both delivering effective conservation measures and managing negative impacts of wildlife on human wellbeing [7]. Therefore, identifying how herring gulls in urban areas respond to human behaviour is likely to be key in developing effective means of mitigating conflict, but has largely been overlooked.

One possible method of lessening problematic behaviour by wildlife is through exploiting natural reactions to perceived threats, such as a sensitivity to gaze. Gaze aversion is the tendency to show a fearful response towards being watched, characterized by avoidance or a slower approach towards a desired object or location [8]. It is thought to be an adaptive anti-predator response across a range of vertebrate taxa [8]. Several bird species show aversion towards human gaze [9–15]. Nesting American herring gulls (*Larus smithsonianus*) fled sooner when experimenters approached their nests directly rather than walking past them [16], indicating that they can respond to subtle differences in human behaviour. However, the authors did not explicitly test for gaze aversion. Here, we exploited a common scenario in coastal towns, where herring gulls approach humans for food, and tested whether herring gull approach behaviour towards food was affected by human gaze direction. We predicted that herring gulls would take longer to approach the food source when an experimenter was looking directly at them rather than looking away.

## 2. Material and methods

### (a) Test subjects

We studied herring gulls (hereafter ‘gulls’) in coastal towns in Cornwall, UK, as these individuals are likely to have experienced anthropogenic food and to be habituated to human presence. We targeted gulls that were not in flight nor engaged in antagonistic interactions. Individual gulls or mated pairs inhabit spatially distinct feeding areas [17], from which they chase away intruders. This, as well as the presence of identifiable gulls, allowed us to avoid mistakenly testing the same individual multiple times.

### (b) Experimental protocol

Experiments were conducted between 16 November and 11 December 2018. We placed 250 g of fried potato chips *ca* 1.5 m (see electronic supplementary material, Methods) in front of the experimenter. The food was presented inside a sealed, transparent freezer bag weighed down with a 550 g weight to prevent gulls from eating the food: rewarding the gull in the first trial might have generated order effects. The experimenter took a crouched position with her body oriented towards the gull to enable a direct line of sight with it once it walked towards the food. The experimenter initially looked intermittently at the gull to locate it, but once in position, she used her peripheral vision to watch for it to approach. When the gull started approaching (either placing a foot forward towards the food or landing with both feet on the ground if starting from an elevated position), the experimenter started a stopwatch and adopted the gaze direction associated with the experimental treatment assigned to the trial.

In the ‘Looking At’ treatment, the experimenter directed her gaze towards the eye(s) of the gull and turned her head, if necessary, to follow its approach path until the gull completed the trial by pecking at the food bag. We counted the number of head movements to control for the possibility that gulls may be responding to head movement alone. In the ‘Looking Away’ treatment, the experimenter turned her head and eyes approximately 60° (randomly left or right) away from the gull and maintained this position until she heard the gull peck at the food bag. If a gull did not touch the food within 300 s of starting its approach but remained in the vicinity, the trial was deemed complete and a time of 300 s was recorded. Only completed trials were included in analyses. We recorded approach times to the nearest second. A second experimenter used a Panasonic HC-V770 video camera mounted on a tripod and placed *ca* 8 m from the main experimenter to capture trials and verify distances and timings.

We adopted a repeated measures design to assess the effect of gaze direction within individuals. We randomly assigned individuals to receive Looking At or Looking Away first, and trial order was counterbalanced across individuals. Second trials commenced 180 s after the completion of the first trial to allow normal behaviour to resume. During this inter-trial interval, we tracked the gull using peripheral vision and concealed the food. Trials in which gulls went out of sight were excluded from the analysis.

We also determined whether gulls that did not approach on the ground during the trials were motivated to consume the food but had been deterred by the experimenter's proximity. To quantify this, we recorded if they (i) had approached from an elevated position but did not land on the ground (thus not meeting the experimental starting conditions) or (ii) approached the packaged food after the 300 s trial within a further 60 s of the experimenter retreating to the camera positioned *ca* 8 m away.

### (c) Statistical analysis

We analysed the data in R v. 3.5.2 [18] using a linear mixed-effects model (LMM) from the package lmerTest [19]. We log-transformed the approach times (response variable) to satisfy the normality assumptions of the model. Fixed effects were treatment (Looking At/Looking Away), the distance between the gull and food at the start of the trial, the distance between the experimenter and food, the presence (i.e. within *ca* 8 m radius of the focal gull) of people (yes/no) and other gulls (yes/no), and trial order (to test for habituation). Gull identity was included as a random effect. We compared this full model with one excluding gaze treatment using a likelihood ratio test (LRT) to test whether including gaze significantly increased the model fit to better explain approach times. As Looking At was associated with an increased number of head movements, we also compared the gaze model with one that, instead of gaze, contained the number of head movements as a fixed effect, using another LRT. An independent observer scored all videos and inter-observer agreement was very high (intraclass correlation coefficient (ICC) for gull approach times (*n* = 38 trials): ICC = 0.99, *p* < 0.001; head movements (*n* = 38 trials): ICC = 0.93, *p* < 0.001)).

To explore inter-individual differences in approach behaviour, we conducted a Spearman's rank correlation to test (i) whether individuals' approach times in Looking At were correlated with approach times in Looking Away, and (ii) whether gulls that took longer to approach in Looking At showed the greatest decrease in approach time during Looking Away.

## 3. Results

We attempted to test 74 herring gulls. Only 27 of these (36%) initiated the start of at least one trial. The remaining gulls either flew away soon after presentation of the food or did not approach on the ground within 300 s. Twenty-three (49%) of the 47 gulls that did not approach during a trial approached the food outside the trial conditions (electronic supplementary material, table S1). Nineteen gulls (26% of all those targeted) completed the paired trials, and the analysis is based on these data.

Gulls took significantly longer to approach the food source when the experimenter looked at them versus away (LMM, effect of gaze in full model: *t*_18_ = 2.27, *p* = 0.037, table 1; LRT, effect of gaze when dropped: $\chi_{8}^{2}=5.41,$ *p* = 0.020; electronic supplementary material, table S2). The median difference in approach time between treatments was 21 s. The effect of experimenter gaze direction was apparent while the model also controlled for the gull's starting distance from the food (LMM, effect of distance: *t*_30_ = 3.33, *p* = 0.002). Gulls also took longer to approach the food when other people and other gulls were present (effect of people: *t*_30_ = 2.78, *p* = 0.009; gulls: *t*_16_ = 4.01, *p* < 0.001). There was no significant effect of experimenter distance to the food (LMM, *t*_18.6_ = −0.69, *p* = 0.500) nor of trial order (*t*_17.5_ = 0.86, *p* = 0.404) on approach time. Gaze direction was a significantly better predictor of the gulls' latency to approach the food than was the number of experimenter head movements (LRT, $\chi_{7}^{2}=2.14,$ *p* < 0.001; electronic supplementary material, table S3). In 10 (53%) of the 19 Looking At trials, the experimenter did not move her head.

**Table 1.** Results of the full LMM of herring gull latency to approach food when the experimenter's gaze was directed at the gull (gaze (Looking At) versus Looking Away), with log approach time (seconds) as the response variable.

fixed effects	estimate	s.e.	d.f.	*t*	*p*-value
intercept	3.03	2.10	19.1	1.44	0.166
gaze (Looking At)	0.650	0.287	17.2	2.27	0.037
distance	0.411	0.123	30.0	3.33	0.002
other gulls (yes)	1.50	0.375	16.0	4.01	0.001
people (yes)	1.16	0.415	30.0	2.78	0.009
experimenter distance	−0.972	1.41	18.6	−0.688	0.500
trial order (2)	−0.247	0.288	17.5	−0.856	0.404
**random effect**	**variance**				
gull identity	0.224				

**Figure 1.**
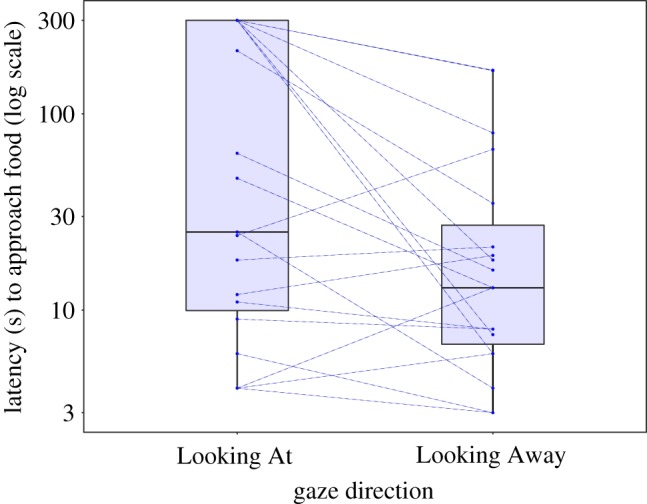
Paired plot of the time taken for individual herring gulls (*n* = 19) to approach a food source when an experimenter was looking at them versus away. Dashed lines indicate within-individual differences in approach time. The majority of individuals took less time to approach when the experimenter's gaze was directed away. (Online version in colour.)

There was large inter-individual variation in time taken to approach the food (Looking At range: 4–300 s; Looking Away range: 3–167 s; figure 1). Six individuals did not touch the food within the 300 s time limit in Looking At, but all touched the food in Looking Away. Individual approach times in Looking At were positively correlated with approach times in Looking Away (Spearman's correlation, *S* = 562.29, *ρ* = 0.54, *n* = 19, *p* = 0.027), but this relationship appears to be principally driven by two individuals with exceptionally long approach times (greater than 150 s in Looking Away; electronic supplementary material, figure S1). Gulls that took the longest time to approach in Looking At showed the largest reduction in approach time in Looking Away (*S* = 188.35, *ρ* = 0.83, *n* = 19, *p* < 0.001; electronic supplementary material, figure S2), suggesting that these individuals were particularly sensitive to human gaze direction.

## 4. Discussion

Interactions between herring gulls and humans are increasingly leading to conflict and may have the potential to exacerbate population declines of this species. Characterizing the nature of these interactions is therefore an important first step in mitigating negative effects on both humans and gulls. We found that the majority of gulls in urban areas would not approach a food source placed in close proximity to a human, despite many displaying interest in the food. Those that did approach were also more hesitant in the presence of other people and gulls. This suggests that most gulls may be too fearful to engage in food-snatching and that this behaviour is likely to be conducted by a select few individuals.

We found that human gaze direction significantly affected gulls' latency to approach the food: gulls took less time to approach when the experimenter was facing away versus looking directly at them. This demonstrates that gulls use behavioural cues from humans when making foraging decisions in urban environments, and that they find human gaze aversive.

Gulls’ approach times varied widely, with some touching the food within 10 s in both treatments, whereas others did not complete their approach when human gaze was directed towards them. The difference in approach time between treatments was largest for those gulls that took the longest time to approach when being watched, indicating variation in the degree to which gulls find human gaze aversive. This may be because of differences in attention towards the experimenter's eyes or head, variation in boldness or cognitive abilities, or through associative learning during previous interactions with humans [8]. If human gaze aversion is a learned response, those individuals that have been chased away from food by humans may learn to associate human eye contact with potential danger. Alternatively, gaze aversion may be present upon hatching, with gulls being able to generalize the salient features of a vertebrate eye [8,20].

Gulls may have taken more time to approach food while being looked at because they can take another's perspective. However, such perspective-taking remains difficult to disentangle from simpler cognitive processes such as associative learning [21]. Gaze aversion, and gaze sensitivity more broadly, occurs in all three amniote classes and, as such, may have deep evolutionary origins [22]. Further work that focuses on differences in gaze sensitivity at the individual, population and species level will improve our understanding of the development and evolution of gaze-mediated behaviour. Additionally, other cognitive mechanisms allowing gulls to adapt to anthropogenic environments may be important in understanding and mitigating conflict between humans and gulls [23].

In summary, our results indicate that the majority of urban herring gulls are unlikely to approach food when humans are nearby. Those gulls that did approach responded to subtle behavioural cues from the experimenter, suggesting that increased vigilance by humans may reduce food-snatching behaviour. Understanding individual variation in behaviour, and responses towards human behavioural cues more generally, may help inform conservation and control strategies for managing conflict between humans and wildlife in a wide range of taxa.

## Supplementary Material

Goumas et al. Supplementary material

## Data accessibility

The data and R code used for the statistical analyses can be found at https://doi.org/10.5061/dryad.3jf80g4 [24].
