# Supplementary material for: Herring gulls respond to human gaze direction
Source: Biol Lett. 2019 Aug 7;15(8):20190405. doi: 10.1098/rsbl.2019.0405 (PMC6731478; doi:10.1098/rsbl.2019.0405)
Supplement: Goumas et al. Supplementary material [file rsbl20190405supp1.docx]

Herring gulls respond to human gaze direction: Supplementary Material
Goumas M, Burns I, Kelley LA, Boogert NJ.

**Supplementary Methods**

Our study sites were in Falmouth (50.2N, 5.07W), Hayle (50.2N, 5.42W), Helston (50.1N, 5.28W), Newquay (50.4N, 5.08W), Penryn (50.2N, 5.12W), Penzance (50.1N, 5.53W), Perranporth (50.3N, 5.16W), Porthleven (50.1N, 5.32W), Portreath (50.3N, 5.29W), St Ives (50.2N, 5.48W) and Truro (50.3N, 5.05W). Exact coordinates for the locations of each of the gulls tested and included in the paired analysis are included in the dataset. We used potato chips (fries) inside a clear bag as bait because this food source is likely to be widely recognised by gulls in coastal towns, and pilot tests showed that they would consistently approach food presented in this manner. We used chalk to mark the placements of the food and experimenter, and measured the distance, to the nearest cm, after each trial to avoid deterring the gull from approaching (i.e. a distance of 1.5 m between the food and experimenter was estimated at the start of the first trial and the exact distance was included in the model in case the experimenter’s proximity affected approach time). In some cases, these marks could be used for positioning in second trials on the same test subject. The same or similar dark clothes were worn by the experimenter for each trial.

To measure the distances between the food and the starting location of the gull, we held an Excelvan laser range finder parallel to the ground and pointed it towards a flat surface where the gull had been positioned at the start of its approach. Approaches were only timed when the gull was level with the food: where gulls began their approach on elevated surfaces, we measured the distance from the position at which the gull landed on the ground. In the cases where gulls began their approach before the experimenter took position, we started the timer, and determined the distance, when the experimenter was in the correct position.

We also determined whether the gulls that did not approach the food and thus did not start an experimental trial had noticed the food and were motivated to try to consume it. Some gulls started an approach towards the food from an elevated surface, such as a wall, but did not come down to the level of the food. Some gulls flew down towards the food and then flew away. These gulls were recorded as not approaching, as they did not meet the criterion of approaching on the ground, but they were recorded as being motivated (i.e. it was assumed that the gull would have continued to approach the food if the experimenter had not been present). For all gulls that did not approach on the ground but remained in the area, the experimenter left the food in place and retreated to establish whether the gulls would approach within 60 s if she was not in close proximity. These data are reported in Table S1 below. It was not possible to determine whether or not those gulls that flew away upon the experimenter taking position, or that did not approach while in close proximity to conspecifics, would otherwise have been motivated to approach the food.

The sex and age of the gulls were recorded where it was possible to do so. Sex was determined by dominance and pair bond behaviour and size differences when present with the mate. Lone gulls and juveniles were recorded as being of unknown sex, with the exception of two particularly large adult individuals with large heads and bills, which were presumed to be male [1]. Age was determined by the plumage and was categorised as first calendar year (1CY), second calendar year (2CY), fourth calendar year (sub-adult) and adult (fifth calendar year or older; no third calendar year birds participated in the trials).

**Supplementary Results**

We were unable to determine the sex of the majority of gulls that participated in our study with certainty. We identified six gulls as being male and one female. Most of the individuals participating in the experiments were adults, with two 1CY, three 2CY and one sub-adult participating. The small sample of juveniles precludes statistical analyses of age effects. Five gulls used in the analysis were present with their mates. Gulls took longer to approach when another gull was present, whether it was their mate or another individual (main text, Table 1). With further study, it may become apparent that sex may explain some of the variation in approach behaviour, but we were unable to test for its effect here.

**Table S1**. The locations and total numbers of the herring gulls that a) were targeted for the experiment, b) did not approach during the trials but approached the food outside the trial conditions, c) approached but did not complete the first (*n* = 6) or the second (*n* = 2) trial, and d) completed both trials (paired treatments).

| **Town** | **a) Targeted** | **b) No approach, motivated** | **c) Approached during one trial** | **d) Completed paired trial** |
| --- | --- | --- | --- | --- |
| Falmouth | 24 | 8 | 0 | 5 |
| Hayle | 6 | 5 | 1 | 0 |
| Helston | 4 | 0 | 1 | 1 |
| Marazion | 3 | 2 | 0 | 0 |
| Mousehole | 2 | 2 | 0 | 0 |
| Newquay | 4 | 1 | 0 | 3 |
| Penryn | 6 | 1 | 3 | 1 |
| Penzance | 4 | 0 | 0 | 2 |
| Perranporth | 1 | 0 | 0 | 1 |
| Porthleven | 7 | 1 | 1 | 0 |
| Portreath | 1 | 0 | 1 | 0 |
| St Ives | 11 | 3 | 1 | 5 |
| Truro | 1 | 0 | 0 | 1 |
| **TOTAL** | **74** | **23** | **8** | **19** |


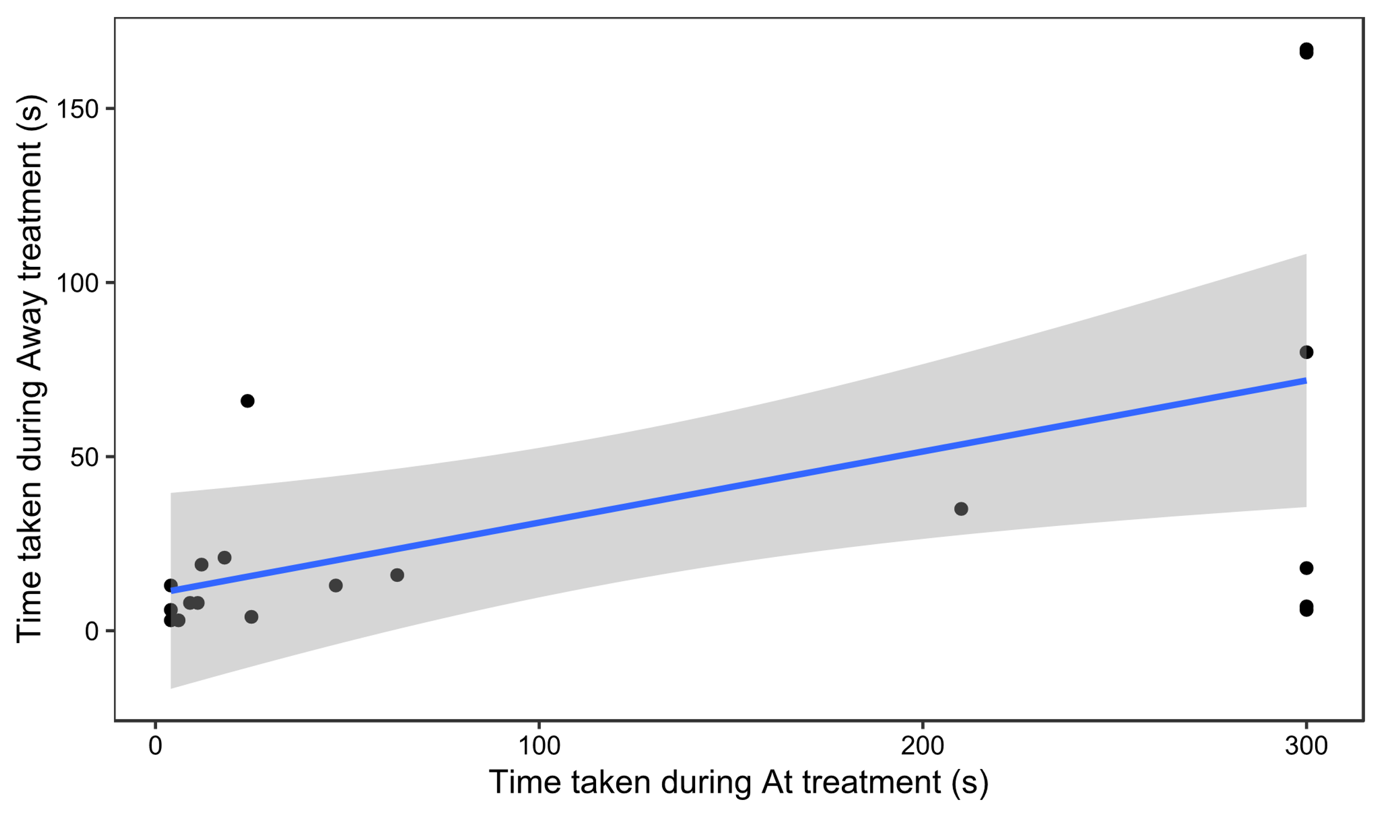


**Figure S1**. Herring gulls appeared to show some repeatability in their latency to approach a food source: individuals that approached slowly when an experimenter directed gaze towards them also tended to approach slowly when gaze was directed away, perhaps suggesting that herring gulls show consistent inter-individual differences in risk-taking behaviour across contexts. The shaded area represents the 95% confidence interval.


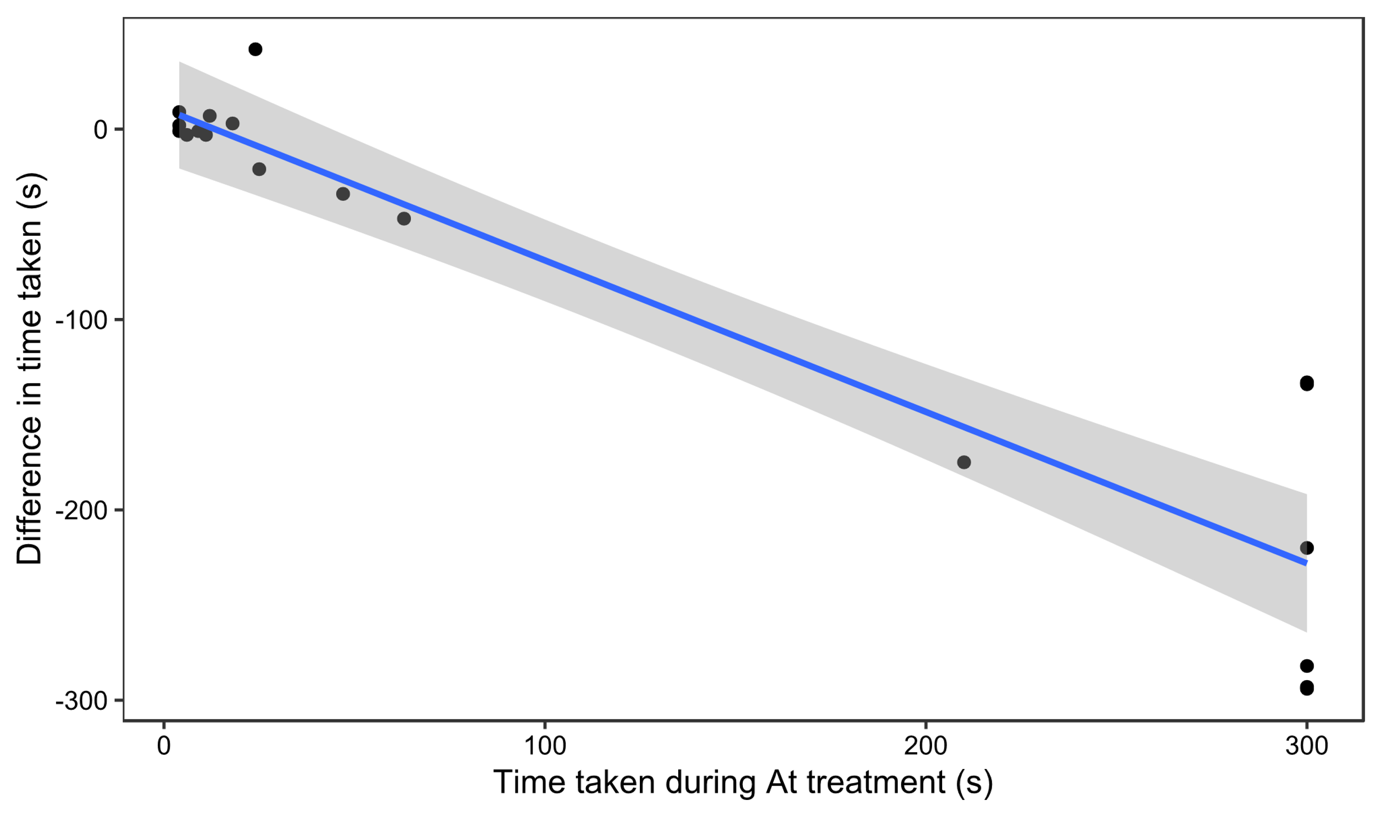


**Figure S2**. Herring gulls that took longest to approach when an experimenter was gazing at them exhibited the largest decreases in approach times when the experimenter was gazing away. The shaded area represents the 95% confidence interval.

**Table S2**. Results of the likelihood ratio test comparing the effect of the addition of gaze treatment to the model. Both models contained the gull’s starting distance from the food, the presence of other gulls, the presence of people, trial order and the experimenter’s distance from the food. Model 1a included gaze treatment (Looking At vs. Looking Away) whereas Model 2a did not.

|  | **DF** | **AIC** | **BIC** | **logLik** | **Deviance** | **ChiSq** | **P-value** |
| --- | --- | --- | --- | --- | --- | --- | --- |
| Model 1a | 9 | 115.63 | 130.37 | -48.81 | 97.63 |  |  |
| Model 2a | 8 | 119.03 | 132.13 | -51.52 | 103.03 | 5.41 | 0.020 |

**Table S3.** Results of the likelihood ratio test comparing the effect of gaze treatment with the effect of head movements. Both models contained gull starting distance from the food, the presence of other gulls and the presence of people. Model 1b featured the number of head movements made by the experimenter as the fixed effect, and Model 2b featured the gaze treatment type. As these two variables were correlated, they were compared in separate models.

|  | **DF** | **AIC** | **BIC** | **logLik** | **Deviance** | **ChiSq** | **P-value** |
| --- | --- | --- | --- | --- | --- | --- | --- |
| Model 1b | 7 | 115.09 | 126.55 | -50.54 | 101.09 |  |  |
| Model 2b | 7 | 112.95 | 124.41 | -49.48 | 98.95 | 2.14 | <0.0001 |

**References**

1. Coulson JC, Thomas C, Butterfield JE, Duncan N, Monaghan P, Shedden C. 1983 The use of head and bill length to sex live gulls Laridae. *Ibis* **125**, 549-557. (doi:10.1111/j.1474-919X.1983.tb03148.x)
